# Causes and Predictability of the Negative Indian Ocean Dipole and Its Impact on La Niña During 2016

**DOI:** 10.1038/s41598-017-12674-z

**Published:** 2017-10-03

**Authors:** Eun-Pa Lim, Harry H. Hendon

**Affiliations:** Bureau of Meteorology, Melbourne, VIC 3001 Australia

## Abstract

In the latter half of 2016 Indonesia and Australia experienced extreme wet conditions and East Africa suffered devastating drought, which have largely been attributed to the occurrence of strong negative Indian Ocean Dipole (IOD) and weak La Niña. Here we examine the causes and predictability of the strong negative IOD and its impact on the development of La Niña in 2016. Analysis on atmosphere and ocean reanalyses and forecast sensitivity experiments using the Australian Bureau of Meteorology’s dynamical seasonal forecast system reveals that this strong negative IOD, which peaked in July-September, developed primarily by the Indian Ocean surface and subsurface conditions. The long-term trend over the last 55 years in sea surface and subsurface temperatures, which is characterised by warming of the tropical Indian and western Pacific and cooling in the equatorial eastern Pacific, contributed positively to the extraordinary strength of this IOD. We further show that the strong negative IOD was a key promoter of the weak La Niña of 2016. Without the remote forcing from the IOD, this weak La Niña may have been substantially weaker because of the extraordinarily long-lasting warm surface condition over the dateline from the tail end of strong El Niño of 2015–16.

## Introduction

The Indian Ocean Dipole mode (IOD) is the leading mode of interannual variability of sea surface temperature (SST) in the tropical Indian Ocean during the boreal summer-autumn seasons^1–3^ (Supplementary Fig. S1). The positive phase of the IOD (+ve IOD) is characterised by negative SST anomalies to the west of Java and Sumatra and positive SST anomalies in the tropical central-western Indian Ocean. Opposite signed SST conditions are found during the negative phase of the IOD (−ve IOD). The IOD typically develops during boreal summer, peaks in autumn, and then rapidly decays in November and December when Australian summer monsoon starts^1,4–6^. The IOD significantly affects the climate of the Indian Ocean rim countries such as eastern Africa, India and Indonesia^1,4,7,8^ and remotely influences the climate of southern Australia and north eastern Asia that are located under the pathways of equivalent barotropic Rossby waves emanating from the tropical Indian Ocean^9–13^.

Development of the IOD is often linked with the El Niño-Southern Oscillation (ENSO) in the Pacific because of variations of the Walker Circulation, which has been explored by a number of studies^3,12–17^. In general, the +ve IOD tends to occur with El Niño (Supplementary Fig. S1), and the −ve IOD with La Niña, although exceptions exist^18^. This relation between the IOD and ENSO peaks in boreal autumn when remotely forced wind anomalies over the Indian Ocean, as a result of the alteration of the Walker Circulation in response to ENSO in the Pacific, are reinforced by strong local positive air-sea feedbacks^4,6^. The remote forcing of the IOD by ENSO is an important source of long-lead predictability of the IOD^17,19^. The IOD may also act to reinforce or weaken concurrent development of ENSO^15,16^ and subsequent development of ENSO in the following year^20^.

**Figure 1:**
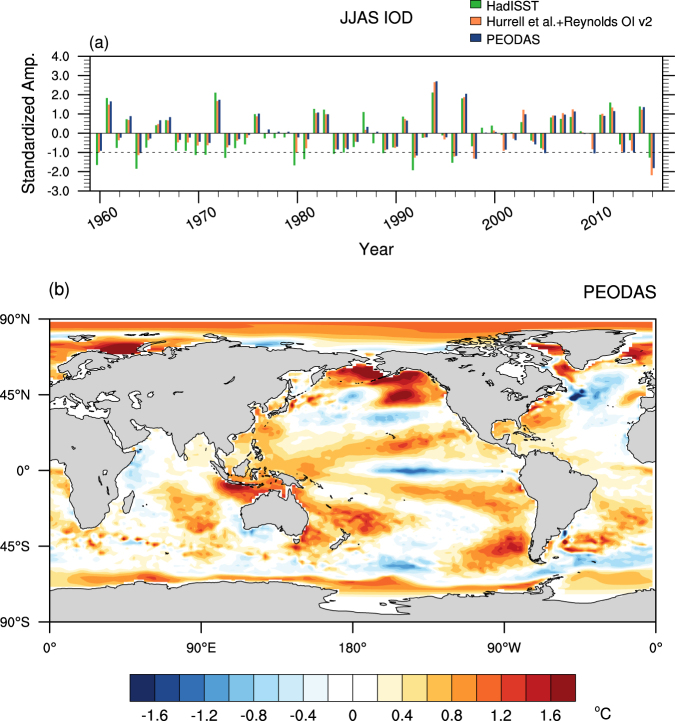
The Indian Ocean Dipole amplitudes and the sea surface temperature anomalies of June to September 2016. (**a**) Standardized Indian Ocean Dipole Mode Index (DMI) obtained with the June to September (JJAS) mean SST anomalies of PEODAS ocean reanalysis (dark blue bars), the merged Hadley-NOAA/OI SST^22–24^ analyses (orange bars), and the HadISST dataset^24^ (green bars) from1960 to 2016. The dotted horizontal line indicates negative 1 standard deviation (σ) of JJAS DMI. The SST anomalies used in the DMI calculation were relative to the climatology of 1981–2010. (**b**) 2016 anomaly pattern of PEODAS JJAS mean SST relative to the JJAS mean climatology of 1981–2010. The colour shading interval is 0.2 °C. Plots were generated using the NCAR Command Language version 6.3.0 (www.ncl.ucar.edu).

The current study is motivated by the extraordinarily rapid and strong development of the −ve IOD in 2016, which was the strongest −ve IOD event observed during June to September in the last 60 years as diagnosed using the POAMA ensemble ocean data assimilation system (PEODAS)^21^ ocean reanalyses and the merged Hadley-NOAA/OI SST^22–24^ analyses (Fig. 1 and Supplementary Fig. S2). Although the −ve IOD of 2016 was not an extreme event as depicted in the HadISST dataset^24^, it was a strong event with a greater than 1 standard deviation (σ) magnitude of the Indian Ocean Dipole Mode Index (DMI^1^; see Methods for details) (Fig. 1 and Supplementary Fig. S2). This strong −ve IOD of 2016 was reported to have played a key role in promoting the extreme wet conditions over Indonesia and Australia from May onwards and was later accompanied by a weak La Niña in the tropical Pacific, which exacerbated drought in East Africa^25^.

In our attempt to understand the causes and predictability of the strong −ve IOD of 2016, we note the following two climate features with particular interest. First, this −ve IOD was possibly pre-conditioned by the record warmth of the tropical Indian Ocean stemming from the tail end of strong El Niño of 2015–16^26,27^ and from the long-term warming trend of the tropical Indian Ocean in boreal spring (0.6 °C warming over 1960–2014 at the 97% confidence level (c.l.); see Methods for the calculation of statistical significance) (Supplementary Fig. S3). Second, despite the extraordinary strength of the −ve IOD and the massive discharge of heat from the equatorial Pacific following the strong El Niño in 2015–16, La Niña in the Pacific in boreal autumn and winter of 2016 was relatively weak^25^. Thus, we explore the causative mechanism and predictability of the −ve IOD of 2016 with focus on i) the role of the long-term warming trend in the ocean for development of this IOD event and ii) the influence of the strong −ve IOD on the unexpectedly weak La Niña of 2016.

Our approach is to conduct forecast sensitivity experiments using the Australian Bureau of Meteorology’s dynamical seasonal forecast system, POAMA^28^. This system has demonstrated skill to predict peak season ENSO and IOD at the lead times of beyond 9 months and up to 5 months, respectively^5,19,29^, and therefore, is a suitable tool to investigate impacts of the IOD on ENSO, and vice versa.

For this study, we conducted seven experiments that differed in ocean initial conditions (Table 1 and Supplementary Figs S4 and S5). For each experiment, POAMA was initialised on 21 April 2016 and produced 11 member ensemble forecasts for the following 7 months (May-November). For the control experiment (CTRL), forecasts were initialised with the observed ocean state of 00 UTC 21 April 2016. To understand the role of a long-term temperature trend in the development of the strong −ve IOD of 2016, an experiment was conducted by removing the linear trend, which was computed over the period 1960–2014 at 00 UTC 21 April, from the 3-dimensional ocean temperature and salinity initial conditions (de-trend experiment, DTRND). The linear trend in the tropical SST computed over the 55 years is significantly positive in the central Indian Ocean and far western Pacific Ocean, reaching up to 1.5 °C/55 years (Supplementary Fig. S4). The trend in the equatorial subsurface temperature is also strongly positive (i.e. deepening of the thermocline) in the eastern Indian Ocean, reaching up to 6 °C/55 years (Supplementary Fig. S5). On the other hand, the temperature trend is positive and negative in the western and eastern Pacific subsurface (+1 °C and −3 °C over 55 years, respectively), and so is the trend in the western and eastern Atlantic subsurface (+1.8 °C and −6 °C over 55 years, respectively). Consequently, the tilt of the thermocline has steepened across the Pacific and Atlantic Ocean subsurface over the period of 1960–2014 on 21 April.

**Table 1:** Design of POAMA forecast sensitivity experiments to ocean initial conditions.

	Ocean			Atmosphere & Land
	Indian	Pacific	Atlantic	
**CTRL** Control	**Observed**	**Observed**	**Observed**	**Observed**
**DTRND** De-trend	Observed without temperature and salinity trends	Observed without temperature and salinity trends	Observed without temperature and salinity trends	
**IO** Indian-Ocean only (70°S-30°N, 30-135°E)	**Observed**	*Climatology of 1981–2010*	*Climatology of 1981–2010*	
**IOAO** Indian-Ocean-Atlantic-Ocean only (eq-70°N, 90°W-100°E and 80°S-eq, 70°W-135°E)	**Observed**	*Climatology of 1981–2010*	**Observed**	
**IOPO** Indian-Ocean-Pacific-Ocean only (85°S-70°N, 30-285°E)	**Observed**	**Observed**	*Climatology of 1981–2010*	
**ClimIO** Climatological Indian Ocean (70°S-30°N, 30-135°E)	*Climatology of 1981–2010*	**Observed**	**Observed**	
**CLIM** Climatological Ocean	*Climatology of 1981–2010*	*Climatology of 1981–2010*	*Climatology of 1981–2010*	

Boldface, normal and *italic* types indicate observed, de-trended observed, and climatological initial conditions at 00 UTC 21 April, respectively. For the DTRND experiment, the linear trends of temperature and salinity were computed at 00 UTC 21 April over 1960–2014. Latitude and longitude information in the parentheses denote the domain where realistic (IO, IOAO, IOPO) and climatological (ClimIO) initial conditions were used.

In order to understand the interaction between the strong −ve IOD and the weak La Niña in the tropical Pacific Ocean, we conducted the following four experiments: the Indian-Ocean only experiment (IO) initialised using realistic ocean initial conditions over the Indian Ocean but using climatological ocean conditions (based on 1981–2010) elsewhere; the Indian-Ocean-Atlantic-Ocean only experiment (IOAO) initialised with realistic ocean initial conditions over the Indian and Atlantic Oceans but climatological ocean conditions elsewhere; the Indian-Ocean-Pacific-Ocean only experiment (IOPO) initialised with realistic ocean initial conditions over the Indian and Pacific Oceans but climatological ocean conditions elsewhere; and the climatological Indian Ocean experiment (ClimIO) initialised with climatological ocean conditions over the Indian Ocean but realistic ocean conditions elsewhere (Table 1 and Supplementary Figs S4 and S5). Lastly, we ran an experiment by initialising forecasts with climatological ocean initial conditions everywhere (CLIM). The CLIM experiment is used as a reference to compute forecast anomalies. In all seven experiments, atmosphere and land were initialised with observed conditions of 00 UTC 21 April 2016. Hence, the experiments were to generate forecast differences caused by the differences in ocean initial conditions.

For observational analyses, we used monthly PEODAS ocean reanalysis^21^ and ERA-Interim atmospheric reanalysis^30^. We analysed anomalies relative to the climatology of the base period 1981–2010.

## Results

### IOD of 2016

**Figure 2:**
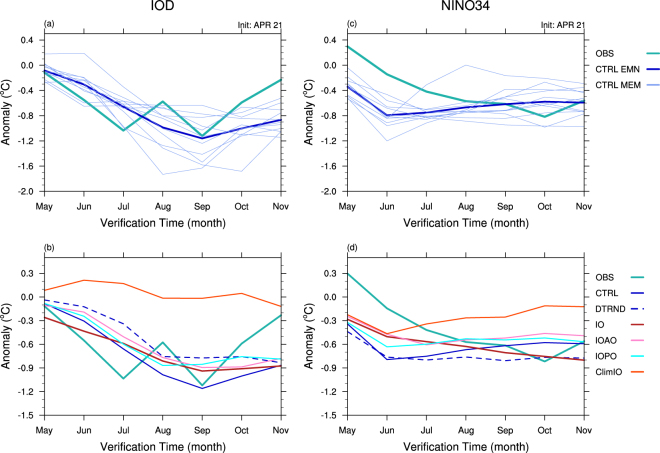
Observed and forecast DMI and NINO3.4 index. Forecasts of the DMI and NINO3.4 index of 2016 initialised on 21 April 2016 from POAMA control experiment (CTRL) (upper panels; **a**,**c**) and five different forecast sensitivity experiments (DTRND, IO, IOAO, IOPO, ClimIO) (lower panels; **b**,**d**). In all four panels, thick green lines denote the indices from PEODAS reanalysis (OBS), and solid thick blue line indicates the 11 member ensemble mean forecasts from CTRL. In (**a**) and (**c**), thin light blue lines indicate individual 11 forecasts from CTRL. In (**b**) and (**d**), dotted blue and solid red, pink, light blue and orange lines indicate the ensemble mean forecasts from the DTRND, IO, IOAO, IOPO and ClimIO experiments, respectively. See Supplementary Figs S6 and S7 for 11 member forecasts of each of the five forecast sensitivity experiments for DMI and NINO3.4 index, respectively. Plots were generated using the NCAR Command Language version 6.3.0 (www.ncl.ucar.edu).

**Figure 3:**
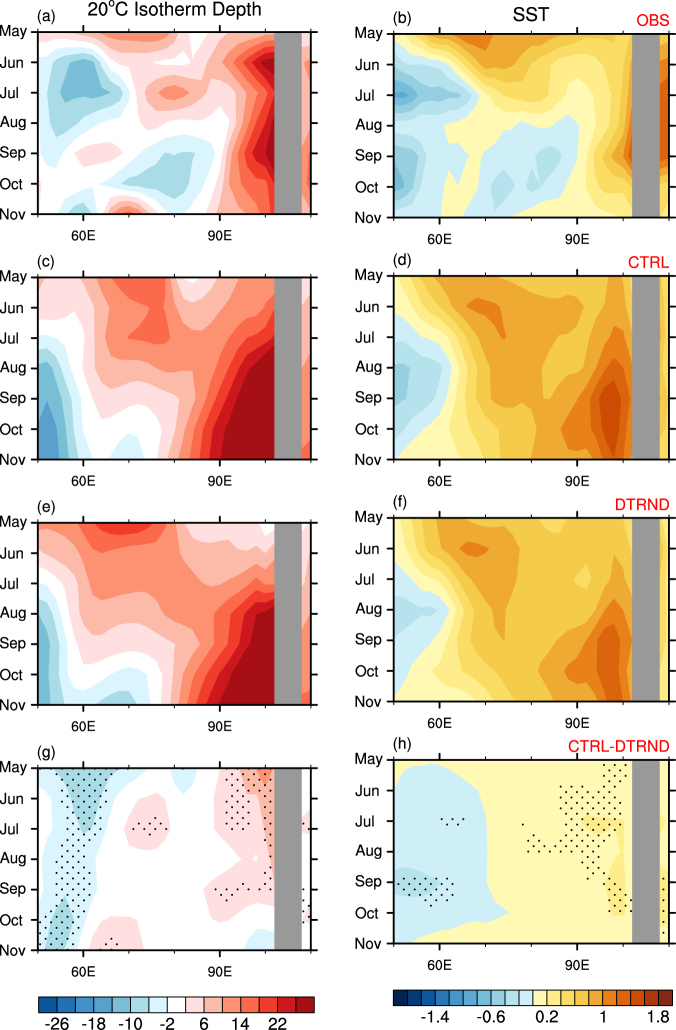
Observed and forecast evolution of monthly anomalies of subsurface and surface temperatures over the southern tropical Indian Ocean from May to November 2016. (left column; **a**,**c**) 20 °C isotherm depth and (right column; **b**,**d**) SST anomalies averaged over 10°S-equator in PEODAS reanalysis (top row; **a**,**b**), CTRL (second row; **c**,**d**) and DTRND (third row, **e**,**f**). Bottom panels (**g**) and (**h**) display the monthly mean differences of 20 °C isotherm depth and SST between CTRL and DTRND, which indicate differences caused by the long-term trends in the ocean temperature initial conditions. Stippling denotes the ensemble mean difference being statistically significant at the 90% c.l., using a two-tailed Student’s t-test with 11 member forecasts of CTRL and DTRND. The colour shading interval is 4 °C and 0.2 °C for the subsurface and surface temperature anoamlies, respectively. Plots were generated using the NCAR Command Language version 6.3.0 (www.ncl.ucar.edu).

The −ve IOD of 2016 reached its maturity in July to September, with the anomalous amplitude greater than 2σ in July and September (Fig. 2a). POAMA control experiment (CTRL) can capture this IOD event as evidenced by the plume of the 11 member ensemble encompassing reality along the course of its evolution. The ensemble mean forecast correctly predicts the maximum strength of the IOD in September despite a substantial degree of uncertainty expressed in the wide spread of ensemble member forecasts (Fig. 2a). In contrast, POAMA falls somewhat short in predicting the rapid development from May to July and fails to predict the rapid termination from September to November. These deficiencies reflect the slow evolution of the forecast cold anomaly in the western pole of the IOD in May to July and the over-predicted warming in the eastern pole of the IOD in September to November, respectively (Figs 2a and 3a–d). Overall, POAMA predicts the monthly evolution of the IOD 2016 event with moderate success as demonstrated by the correlation of 0.53 between the observed and the ensemble mean forecast IOD along the course of 7 month verification period (statistically significant at the 90% c.l. by a one-tailed Student’s t-test).

The de-trend experiment (DTRND) also predicts the occurrence of this −ve IOD (Figs 2b and 3e,f). However, DTRND predicts its strength to be substantially weaker than CTRL because the subsurface and surface temperature anomalies are smaller in both eastern and western poles of the IOD from the beginning of the DTRND forecast, and those differences are maintained in the following months (Fig. 3e–h). The sea surface warming in the eastern pole is significantly weaker in DTRND than CTRL from the beginning of the forecast, which leads to the significantly weaker IOD in DTRND than CTRL at the 95% c.l. during June to October. These results suggest that the underlying positive trend in upper ocean temperatures in the tropical eastern Indian Ocean in late April is likely to have played a non-negligible role for the strength of the −ve IOD of 2016.

Comparisons among the IO, IOAO, IOPO and ClimIO experiments reveal that this IOD is likely to have developed primarily from existing conditions in the Indian Ocean and was not strongly dependent on remote forcing from the Pacific or Atlantic Ocean (Fig. 2b). In particular, the ensemble mean forecast from the IO experiment initialised with the realistic information only over the Indian Ocean can reproduce development of the −ve IOD although its strength for the peak in September is modestly weaker than that of CTRL. Including the realistic Atlantic or Pacific Ocean information in the initial conditions (IOAO, IOPO) makes little difference to the IOD forecasts from the IO experiment. The ClimIO experiment confirms the dominant role played by the Indian Ocean conditions in the development of this IOD event: without realistic Indian Ocean information in the initial conditions, the −ve IOD is not reproduced at all.

In addition, there may have been an interaction among the three ocean basins that boosted the second peak of the IOD in September, given that CTRL demonstrates the best skill to predict it, compared to the IO, IOAO and IOPO experiments (Fig. 2b).

### ENSO of 2016

In contrast to the rapid and strong development of the IOD in boreal summer, the observed cooling over the tropical central to eastern Pacific associated with La Niña during 2016 was moderate (Fig. 2c): the NINO3.4 SST anomalies stayed weaker than 1σ for the 2^nd^ half of 2016. The peak cooling of the tropical Pacific in September-November is skilfully captured by CTRL, but POAMA significantly overestimates the early stage of La Niña development from May to July. The inability of POAMA to predict the gradual growth of this La Niña is reflected in a weak correlation of 0.42 between the observed and the ensemble mean forecast NINO3.4 SST anomalies along the course of 7 month verification period, which does not pass a one-tailed Student’s t-test for the 90% c.l. DTRND predicts the tropical eastern Pacific SSTs to be slightly cooler than CTRL for boreal autumn (Fig. 2d), which is likely explained by the cooling trend in the thermocline in the equatorial eastern Pacific (Supplementary Fig. S5). However, the difference in the NINO3.4 SST between DTRND and CTRL is not statistically significant at the 90% c.l.

The IO, IOAO, IOPO and ClimIO experiments suggest that this weak La Niña condition was largely promoted by Indian Ocean processes (Fig. 2d). The ensemble mean forecast of the IO experiment, which is initialised with observed conditions only in the Indian Ocean, produces La Niña conditions in the Pacific close to the observed: cold biases in the early stage of the forecast are smaller, and magnitudes of predicted cooling from August are realistic, compared to the forecast of CTRL (Fig. 2d). Including observed Atlantic Ocean information to the initial conditions (IOAO) appears to weaken the subsequent La Niña evolution, compared to using observed Indian Ocean information only, but the difference between the IO and IOAO experiments is not statistically significant at the 90% c.l. Interestingly, adding observed Pacific Ocean information to the initial conditions (IOPO) also works against the growth of cooling over the tropical eastern Pacific, compared to using observed Indian Ocean initial conditions only. On the other hand, the forecast initialised with realistic conditions everywhere except over the Indian Ocean (ClimIO; i.e. no realistic Indian Ocean information in the initial conditions) predicts the initial cooling of NINO3.4 SST for May and June, similar to the forecasts of the IO, IOAO and IOPO experiments, but fails to sustain the La Niña cooling. The difference in NINO3.4 SST between the IO and ClimIO experiments is statistically significant at the 99% c.l. from August onwards, and this contrasting evolution of NINO3.4 SST in the IO and ClimIO experiments highlights that Indian Ocean processes were critical to keeping the tropical eastern Pacific cooling in the 2^nd^ half of 2016.

So, why was the −ve IOD of 2016 so strong (and predictable), how did the IOD act to promote La Niña, but why was La Niña so weak?

### Tug of war between strong −ve IOD and long-lasting El Niño

**Figure 4:**
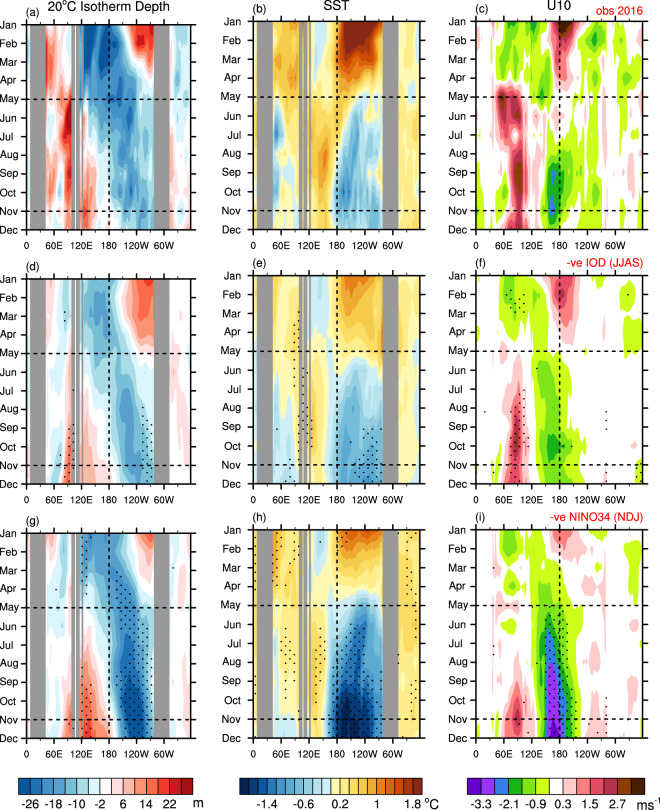
Comparison of 2016 evolution of subsurface and surface temperature and low-level zonal wind anomalies to those of strong negative IOD and La Niña events. Monthly anomalies of (left column; **a**,**d**,**g**) 20 °C isotherm depth, (middle column; **b**,**e**,**h**) SST and (right column; **c,f,i**) 10 m zonal winds averaged over 5°S–5°N for 2016 (top row; **a**–**c**) and for the composites of five strong −ve IOD events of June to September (1992, 1996, 1998, 2005, 2010) (middle row; **d**–**f**) and four strong La Niña events of November to January (1988–89, 1998–99, 2007–08, 2010–11) (bottom row; **g**–**i**) since 1980. Stippling in (**d**–**i**) indicates the composites being statistically significantly different from zero at the 95% c.l. using a two-tailed Student’s t-test. The dateline and May and November are marked by dotted vertical and horizontal lines, respectively, to assist the comparison with the forecasts shown in Fig. 6. The colour shading interval is 4 m, 0.2 °C and 0.6 ms^−1^ for 20 °C isotherm depth, SST, and 10 m zonal winds, respectively. Plots were generated using the NCAR Command Language version 6.3.0 (www.ncl.ucar.edu).

To address these questions, we explore the evolution of the 2016 event and contrast it to the typical evolution for other strong IOD and La Niña events. Figure 4 displays the monthly anomalies of 20 °C isotherm depth, SST and low-level zonal winds averaged over 5°S–5°N for 2016 (top panels) and for the composites of all strong −ve IOD events (middle panels) and all strong La Niña events (bottom panels) that occurred during 1981–2015. The strong −ve IOD events were based on the DMI exceeding −1σ for June-September (1992, 1996, 1998, 2005, 2010), and the strong La Niña events were based on NINO3.4 values exceeding −1σ for November–January (1988–89, 1998–99, 2007–08, 2010–11). For the selection of the strong La Niña events, we also imposed a condition that the SST state of the tropical eastern Pacific in the previous year should be warmer than normal in order to sample the La Niña cases whose pre-condition was similar to that of 2016.

**Figure 5:**
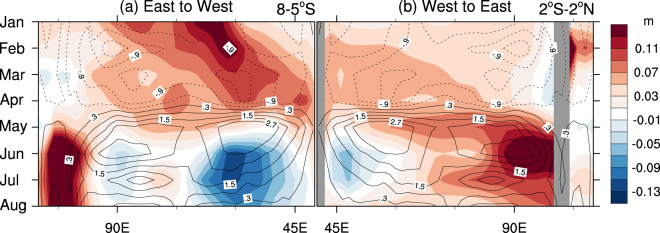
Evolution of (**a**) off-equatorial and **(b**) equatorial sea surface height anomalies in the tropical Indian Ocean during 2016. The colour shading indicates monthly anomalies of sea surface height averaged over the off-equatorial region (8–5°S) and the equatorial region (2°S–2°N). The overlayed contours denote 10 m zonal winds averaged over the equatorial region. The longitude is flipped in (**a**) to highlight the westward propagating off-equatorial Rossby wave that hits the western boundary in April and reflects as an equatorial Kelvin wave that propagates to the east as shown in (**b**). The colour shading interval is 0.02 m, and the contour interval is 0.6 ms^−1^. Plots were generated using the NCAR Command Language version 6.3.0 (www.ncl.ucar.edu).

The −ve IOD of 2016 during June-October was proceeded in January-April by basin-wide warming (Fig. 4b) and easterly anomalies (Fig. 4c) across the equatorial Indian Ocean in association with the strong El Niño and accompanied +ve IOD of 2015–16^6,26,31,32^. These easterly anomalies acted to drive a downwelling westward propagating Rossby wave, which built up subsurface heat at the western boundary by April (Figs 4a and 5a). A combination of reflection of the Rossby wave at the western boundary and relaxation of the easterly anomalies in April appears to have resulted in a strong downwelling Kelvin wave that traversed the basin and arrived in the eastern Indian Ocean in May-June (Figs 4a and 5b). This downwelling Kelvin wave propagation acted to warm the eastern Indian Ocean, and a strong Bjerknes feedback was instigated with the development of westerly anomalies in the central Indian Ocean from May onwards (Fig. 4a–c).

Similar evolutions of SST and zonal wind anomalies are found along the equatorial Indian Ocean in the composites of previous five strong −ve IOD events of June to September (Fig. 4e,f). However, commencement of the negative dipole in association with an arrival of the downwelling Kelvin wave and/or a burst of westerly anomalies in the tropical central Indian Ocean is not obvious in the IOD composite (Fig. 4d,f). Rather, development of the composite IOD events is more gradual through boreal summer. In fact, among the five previous strong −ve IOD cases, only the 1998 event developed with anomalous ocean subsurface heat accumulated in the tropical western Indian Ocean and a surface westerly wind burst in the tropical central Indian Ocean, similar to those of 2016 event but with weaker magnitudes (Supplementary Fig. S8). Thus, the rapid development of the 2016 IOD appears to be an outcome of an optimal combination of the preceding ocean subsurface dynamics that resulted from a strong build-up of heat in the western Indian Ocean and subsequent Bjerknes feedback occurring in the tropical Indian Ocean. This understanding is consistent with the reproducibility of the strong −ve IOD shown in the IO experiment with the realistic initial conditions only over the Indian Ocean.

In comparison, the general evolution of the Indian Ocean during strong La Niña events (Fig. 4g–i) is characterised by the strong and persistent easterly anomalies across the equatorial central Pacific from boreal spring. However, development of the coupled dipole response in the Indian Ocean does not commence until boreal autumn. Shinoda *et al*.^3^ also show that the IOD events associated with ENSO conditions in the Pacific tend to start later in the seasonal cycle than do the IOD events that are largely independent of ENSO.

In the tropical Pacific, the strong El Niño of 2015–16 appears to have set favourable conditions for the development of La Niña in 2016: the discharge of heat after the peak of El Niño resulted in exceptionally strong cold anomalies in the subsurface of equatorial Pacific during early 2016 (Fig. 4a). However, the strong El Niño of 2015–16 lingered extraordinarily long into 2016 (Fig. 4b and Supplementary Fig. S9), with strong westerly anomalies around the dateline persisting well into boreal spring of 2016 (Fig. 4c and Supplementary Fig. S9). As a result, air-sea coupling associated with La Niña did not start until July, and the cooling over the tropical Pacific ended up to be moderate (Fig. 4b,c). Therefore, the long-tail of El Niño of 2015–16, especially over the tropical central Pacific and resultant delay of the air-sea positive feedback appear to be responsible for the development of only weak La Niña despite favourably strong antecedent subsurface conditions. Unlike the La Niña of 2016, the composite of the previous four strong La Niña events shows well established strong Bjerkness feedback between low-level wind and SST anomalies in the tropical Pacific commencing from June (Fig. 4h,i).

**Figure 6:**
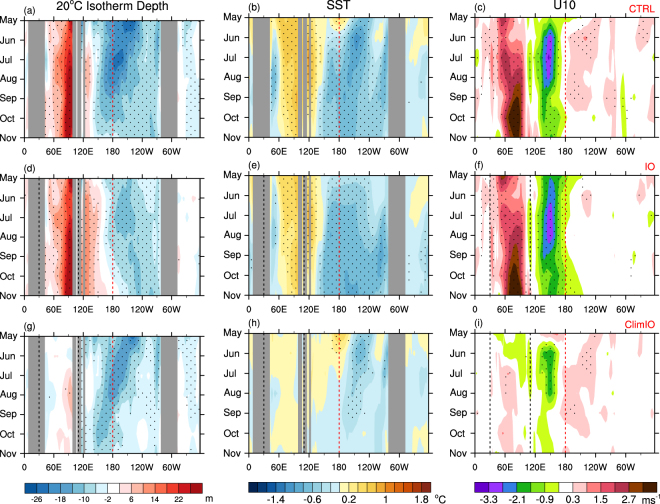
Forecast evolution of monthly anomalies of subsurface and surface temperatures and low-level zonal winds in the equatorial region. (left column; **a**,**d**,**g**) 20 °C isotherm depth, (middle column; **b**,**e**,**h**) SST, and (right column; **c,f,i**) 10 m zonal winds averaged over 5°S–5°N for May to November 2016 from the CTRL (top row; **a–c**), IO (middle row **d–f**) and ClimIO (bottom row; **g**–**i**) experiments. Stippling indicates the forecasts of the CTRL, IO and ClimIO experiments being statistically significantly different from the forecasts of the CLIM experiment at the 95% c.l., using a two-tailed Student’s t-test with 11 member forecasts of each experiment. The red dotted line in (**a**–**i**) indicates the dateline. The black dotted lines in (**d**–**i**) indicate the boundaries where realistic and climatological Indian Ocean initial conditions are used in the IO and ClimIO experiments, respectively. The colour shading interval is 4 m, 0.2 °C and 0.6 ms^−1^ for 20 °C isotherm depth, SST, and 10 m zonal winds, respectively. Plots were generated using the NCAR Command Language version 6.3.0 (www.ncl.ucar.edu).

The ensemble mean forecast from CTRL initialised on 21 April 2016 skilfully predicts the overall characteristics of the evolution of the subsurface and surface temperature anomalies of the tropical Indo-Pacific for the 7 month verification period (Fig. 6a–c). For instance, the model reasonably well captures not only the magnitude of the warm and cold anomalies in the tropical eastern Indian Ocean and the tropical eastern Pacific Oceans, respectively, but also the tendency of westward propagation of the maximum SST anomaly over the tropical Pacific, which is a canonical feature of La Niña^33^ (Fig. 6b compared to Fig. 4b,h). However, the CTRL ensemble overestimates the cooling over the NINO3.4 region in May to July with an unrealistic westward expansion of cold SST anomalies, which is coupled to the forecast easterly bias west of the dateline during boreal summer (Fig. 6b,c). Consequently, CTRL significantly underestimates the warm SST anomalies in the western Pacific from the beginning of the forecast.

The ClimIO experiment (Fig. 6g–i) with realistic ocean initial state everywhere except over the Indian Ocean (i.e. realistic ocean initial state over the Pacific Ocean) is able to reproduce the extraordinarily long-lasting warm SST anomalies over the dateline and its subsequent westward migration and the early stage evolution of La Niña 2016. However, the ultimate outcome is no further development of La Niña, confirming our finding that long-lasting warm SST anomalies over the dateline and resultant delay of the air-sea coupling work against the development of La Niña. On the other hand, the IO experiment (Fig. 6d–f) with realistic ocean initial state *only* over the Indian Ocean (i.e. strong −ve IOD is present but the anomalous sea surface warming over the dateline is not) simulates the evolution of atmosphere and ocean that resembles the canonical strong La Niña shown in Fig. 4g–i. Therefore, our forecast experiments indicate that the strong −ve IOD was the key driver of the development of the weak La Niña condition over the tropical Pacific during the latter half of 2016, which, otherwise, may not have eventuated as it did due to the later than normal termination of El Niño 2015–16 over the tropical central Pacific.

## Summary and Discussion

This study was motivated by the extraordinarily rapid and strong development of the negative phase of the IOD in June to September 2016, which brought extreme heavy rainfall to Indonesia and Australia^25^. This IOD event was later accompanied by a weak La Niña in the tropical Pacific, which contributed to the prolonged drought in East Africa^25^. The record strength negative IOD appears to have been pre-conditioned by the record warmth of the tropical Indian Ocean stemming from the long tail of the strong El Niño during 2015–16 and the long-term warming trend of the tropical Indian Ocean in boreal spring. The weaker than expected La Niña that developed in 2016 was puzzling, given the apparently favourable conditions set by strong subsurface discharge of heat in the tropical Pacific after the strong El Niño of 2015–16. Furthermore, the easterly anomalies in the western Pacific driven by the strong −ve IOD would also have favoured strong La Niña development in the Pacific. Thus, this study strived to uncover the causes and predictability of the strong negative IOD event, including possible contributions from on-going warming trends, and a possible role of this IOD for the development of weaker-than-expected La Niña conditions in the Pacific. Our analysis revealed:

- occurrence and main features of the strong −ve IOD and the weak La Niña of 2016 were skilfully predictable from late April 2016 by the POAMA CTRL run;
- the record strong −ve IOD event in June to September 2016 was promoted by the ocean subsurface and surface dynamics primarily within the Indian Ocean;
- the long-term trend over the last 55 years in sea surface and subsurface temperatures in the Indian Ocean in late April, which is characterised by strong warming over the central Indian Ocean and deepening of the thermocline in the tropical eastern Indian Ocean, contributed to the extraordinary strength of this IOD event;
- there may have been a synergistic interaction among the three ocean basins that strengthened the −ve IOD in September;
- the strong −ve IOD was a key driver of the weak La Niña of 2016 that developed in the tropical Pacific. Without the −ve IOD, the weak La Niña may have been substantially weaker despite the encouraging subsurface conditions set in the 1^st^ half of 2016 by the preceding 2015–16 El Niño;
- the late demise of El Niño of 2015–16 over the tropical central Pacific disrupted the air-sea coupling that was required for the early stage of La Niña development

While the IOD has been the focus of research since the pioneering work of Saji *et al*.^1^, the focus has been strongly skewed toward the +ve IOD for several good reasons: +ve IOD events tend to be stronger and more frequent than −ve IOD events in the post-satellite era^12,34,35^; and a +ve IOD-like long-term trend in the Indian Ocean has been observed in boreal autumn and is projected for the future climate with increasing anthropogenic forcings^36–38^. On the other hand, our study showed that the sea surface has been warming with a local maximum in the central Indian Ocean, and the thermocline has been deepening in the equatorial eastern Indian Ocean as a result of the long-term trend in the mean state of boreal late spring. Such mean state change was found to be able to accelerate and strengthen the development of −ve IOD in the boreal summer of 2016, which was instigated by the downwelling Kelvin wave propagation across the equatorial Indian Ocean. This finding is consistent with those of earlier studies^39,40^ that development of −ve IOD is more favoured than its +ve counterpart when the thermocline of the mean state in the eastern Indian Ocean is deeper than normal. Whether the recent trend in the Indian Ocean is indicative of internal decadal variability that may change its sign in the future or is indeed a reflection of an on-going forced response remains to be determined.

Also, it is a common understanding that the co-variability of ENSO and IOD benefits prediction of the IOD because ENSO drives a favourable teleconnection over the Indian Ocean to promote the IOD, and this ENSO forcing has much longer-lead predictability than the IOD^19^. However, Luo *et al*.^16^ reported that the extreme +ve IOD of 1994 was likely to have caused the moderate El Niño in the tropical Pacific via perturbation of the Walker Circulation. Our result suggests that this could also be true for the –ve IOD. Thus, the frequency of such IOD-driven ENSO events in the historical records and its change in the future climate will be worth exploring in a future study.

Lastly, we found the long-lasting El Niño of 2015–16 worked against the development of strong La Niña, but it was out of the scope of this study to explore why this El Niño lingered so long. Addressing this question may provide useful insights for the predictability of La Niña.

## Methods

### Model and Forecast Generation

POAMA (Predictive Ocean Atmosphere Model for Australia)^28^ is an atmosphere-ocean coupled system, which is run operationally in the Australian Bureau of Meteorology for sub-seasonal to seasonal climate outlook. It consists of the Bureau’s Atmosphere Model version 3 (T47/L17)^41^ and the Australian Community Ocean Model version 2 (2° longitude by 0.5–1° latitude from the tropics to the pole)^42^ coupled by OASIS^43^. Forecasts are initialised with realistic atmosphere, land and ocean conditions generated from the Bureau of Meteorology’s atmosphere and land initialisation scheme (ALI)^44^ and POAMA ensemble ocean data assimilation system (PEODAS)^21^, respectively. PEODAS runs daily and assimilates available subsurface profiles of temperature and salinity, while strongly constraining SST to the Reynolds OI v2 analyses^23^. For each of the seven experiments conducted for this study, an 11 member ensemble was generated using ocean and atmosphere perturbations produced by a coupled-model breeding scheme^44^.

One may question whether the results may be model-dependent and/or be sensitive to the initialisation date. We believe that the credibility of our findings is not significantly limited by the sole use of POAMA because i) ENSO and IOD predictions on seasonal time scale strongly depend on ocean initial conditions^45^ and ii) POAMA’s ability to reproduce observed ENSO and IOD events has been demonstrated with its hindcasts^19,28^ and in our CTRL experiment. Also, to check whether the findings are dependent on the date of initialisation, we repeated the same forecast experiments but initialised on 21 May 2016 and found similar conclusions.

### SST Indices

The strength of the IOD was measured with DMI^1^, which is the difference of the area-averaged SST anomalies in the western pole (10°S–10°N, 50–70°E) and the eastern pole (10°S-eq, 90–110°E). ENSO was monitored by the NINO3.4 index, which is the area-averaged SST anomaly over the NINO 3.4 region (5°S–5°N, 190–240°E).

### Statistical significance test

A two-tailed Student’s t-test was used for testing statistical significance i) on the trends of SSTs averaged over the tropical Indian Ocean in the March-May season (Supplementary Fig. S3) and of ocean surface and subsurface temperature initial conditions at 00 UTC 21 April (Supplementary Figs S4 and 5), given 53 degrees of freedom with 55 samples of 1960–2014; ii) on the composite analysis of strong −ve IOD and La Niña events, given five and four cases, respectively (Fig. 4); and iii) on the ensemble mean difference between two POAMA experiments, given 11 forecasts in each experiment (Figs 2b,d,3 and 6). A one-tailed Student’s t-test was used to estimate the statistical significance on the correlation (*r*) between the observed and forecast monthly evolution of the −ve IOD and NINO3.4 SST index, given 7 samples in each of observed and forecast time series (Fig. 2a,c) (: *r* ≤ 0; : *r* > 0).

### Data availability statement

The datasets generated and/or analysed during the current study are available from the corresponding author on reasonable request.

## Electronic supplementary material

Supplementary Information
