# Supplementary material for: Causes and Predictability of the Negative Indian Ocean Dipole and Its Impact on La Niña During 2016
Source: Sci Rep. 2017 Oct 3;7:12619. doi: 10.1038/s41598-017-12674-z (PMC5626717; doi:10.1038/s41598-017-12674-z)
Supplement: Supplementary file 1 — Supplementary Information [file 41598_2017_12674_MOESM1_ESM.pdf]

Supplementary Information

Causes and Predictability of the Negative Indian Ocean Dipole and Its Impact on La Niña During 2016

Eun-Pa Lim[*] and Harry H. Hendon

Bureau of Meteorology, Melbourne VIC 3001, Australia

[*] Correspondence to e.lim@bom.gov.au

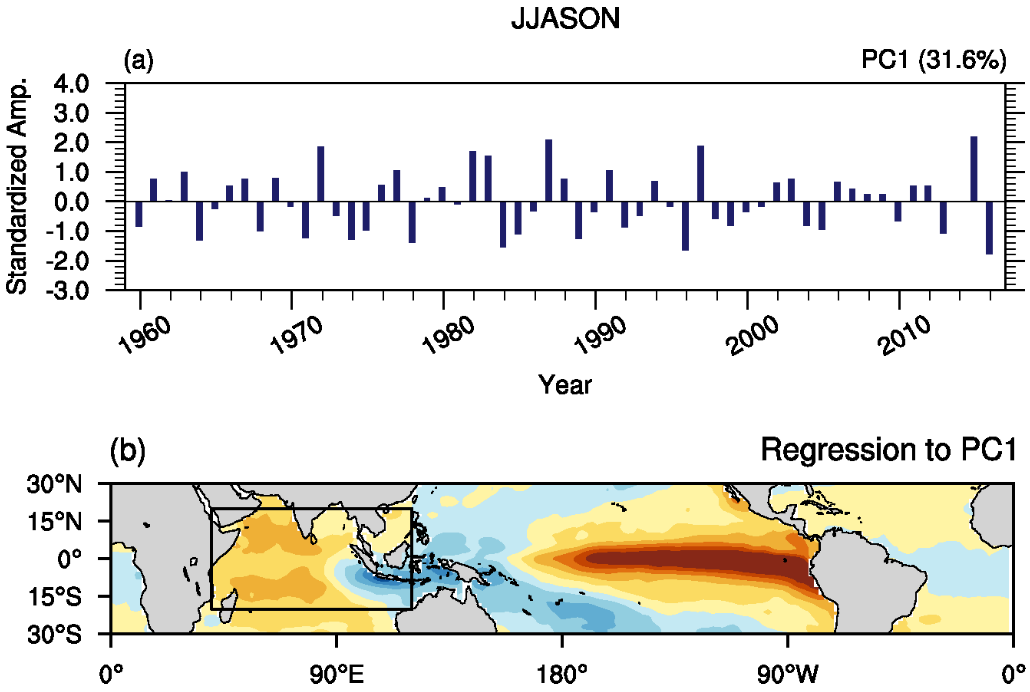

Supplementary Figure S1. The leading mode of interannual variability of the tropical Indian Ocean SST of June to November. (a) Time series of the first mode of the principal component (PC) analysis[1] applied onto the de-trended June to November mean tropical Indian Ocean SST anomalies (20°S-20°N, 40-120°E; the boxed region in (b)) for the period of 1960-2016. (b) Regression pattern of de-trended tropical SST anomalies of the period of 1960-2016 onto the PC time series displayed in (a). PEODAS ocean reanalysis data[2] were used for this PC analysis, and the SST anomalies were relative to the climatology of 1981-2010. Plots were generated using the NCAR Command Language version 6.3.0 (www.ncl.ucar.edu).

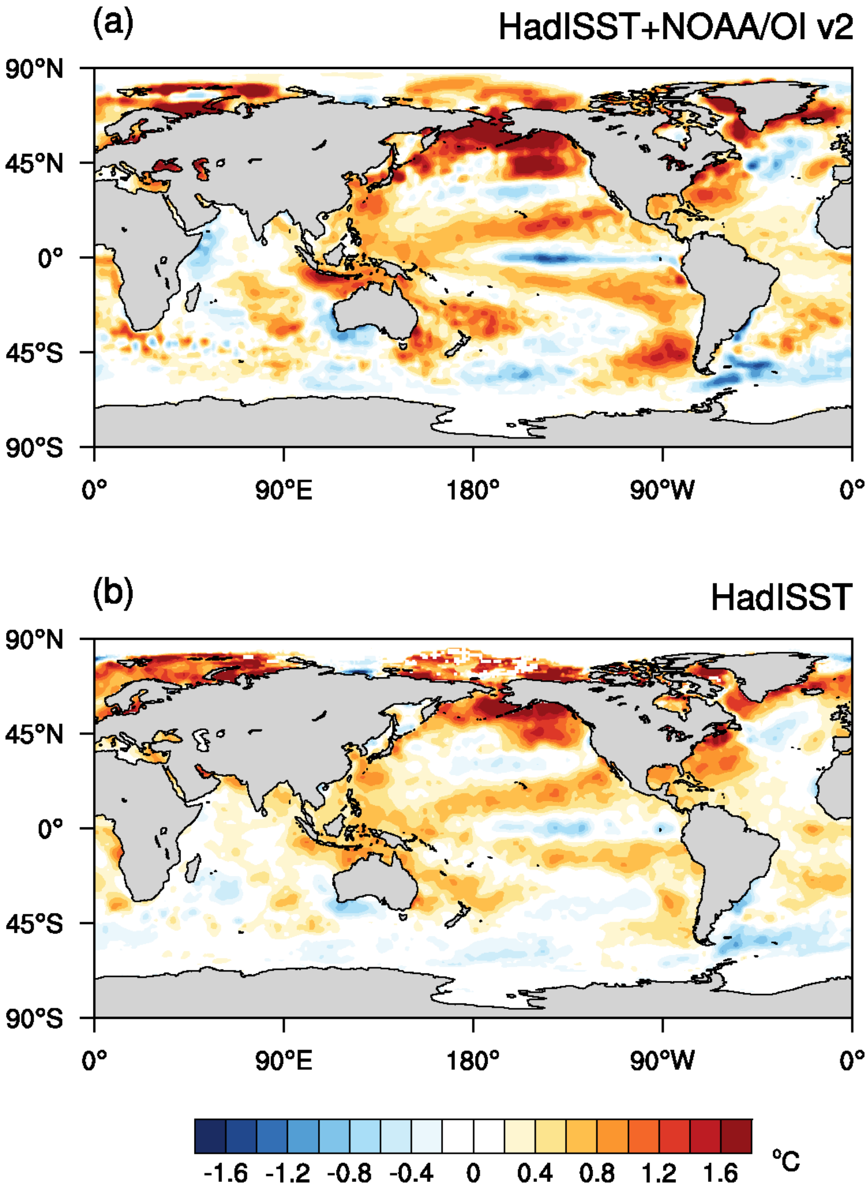

Supplementary Figure S2. 2016 anomaly patterns of June to September mean (JJAS) SST. (a) The merged SST product of HadISST-NOAA/OI SST[3,4,5] and (b) the HadISST dataset[3]. The anomalies displayed in (a) and (b) were relative to their respective JJAS climatologies of 1981-2010. Plots were generated using the NCAR Command Language version 6.3.0 (www.ncl.ucar.edu).

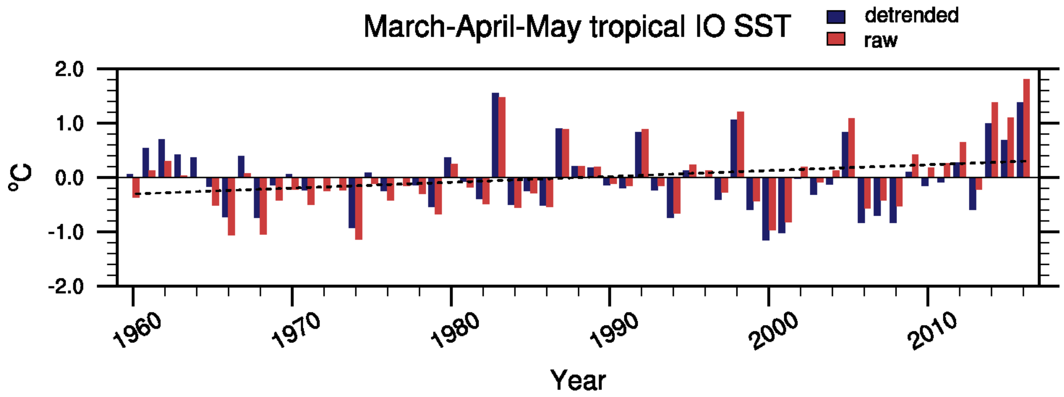

Supplementary Figure S3. March-April-May mean SST anomalies averaged over the tropical Indian Ocean. The SST anomalies of PEODAS ocean reanalysis were averaged over the domain of 20°S-20°N,40-120°E with (red bars) and without (dark blue bars) the long-term linear trend computed over the period 1960-2014. The dotted line indicates the trend (0.6°C over the 55 years, which is statistically significant at the 97% confidence level (c.l.), assessed by a two-tailed Student's t-test with 53 degrees of freedom). Plots were generated using the NCAR Command Language version 6.3.0 (www.ncl.ucar.edu).

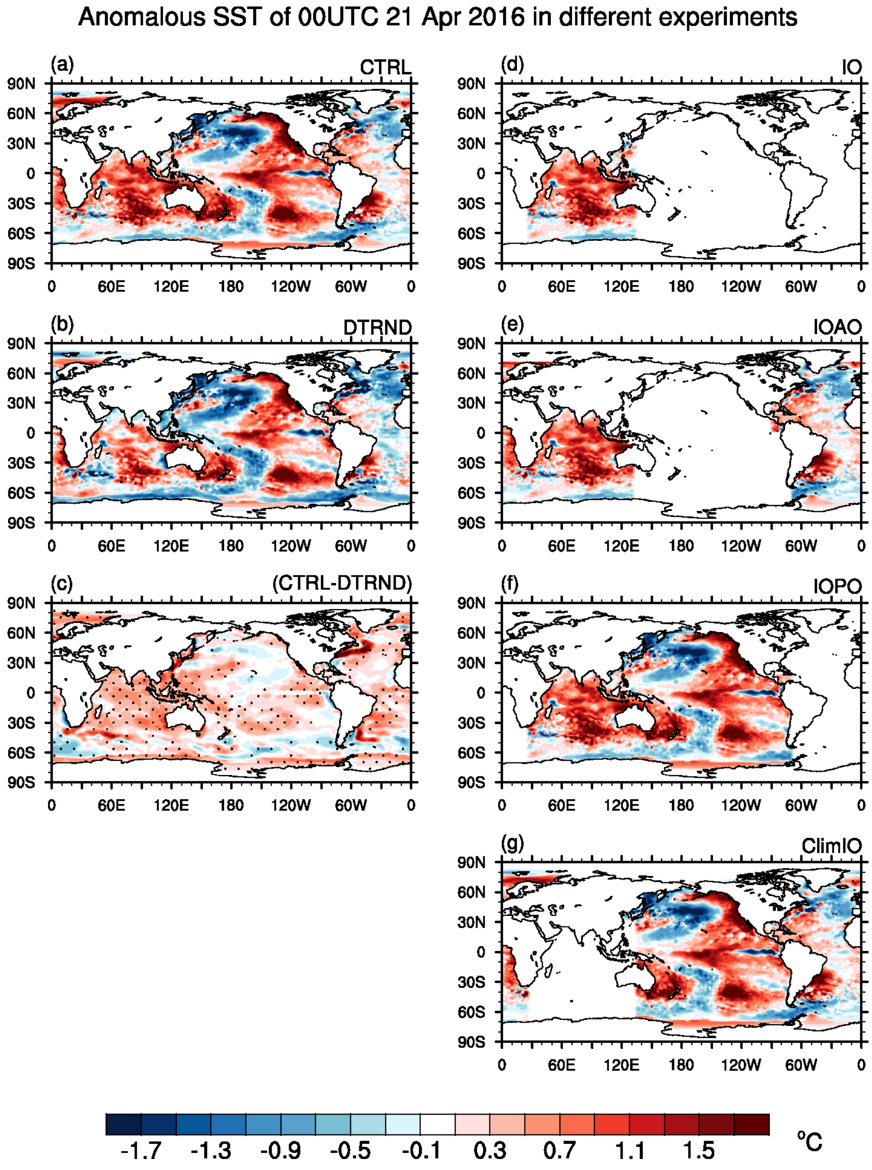

Supplementary Figure S4. Anomaly patterns of PEODAS SST at 00 UTC 21 April 2016, indicating the different SST initial conditions used for the six forecast experiments. (a) Control (CTRL), (b) de-trend (DTRND), (d) Indian-Ocean only (IO), (e) Indian-Ocean-Atlantic-Ocean only (IOAO), (f) Indian-Ocean-Pacific-Ocean only (IOPO) and (g) climatological Indian Ocean (ClimIO) experiments. (c) Difference between (a) and (b) represents the trend estimated over the 1960-2014. Stippling indicates the statistical significance of trend at the 99% c.l. assessed by a two-tailed Student's t-test with 53 degrees of freedom. The SST anomalies displayed here were computed from one set of 3-dimentional ocean initial conditions while 11 sets were used to generate 11 member ensemble forecasts for each experiment. Plots were generated using the NCAR Command Language version 6.3.0 (www.ncl.ucar.edu).

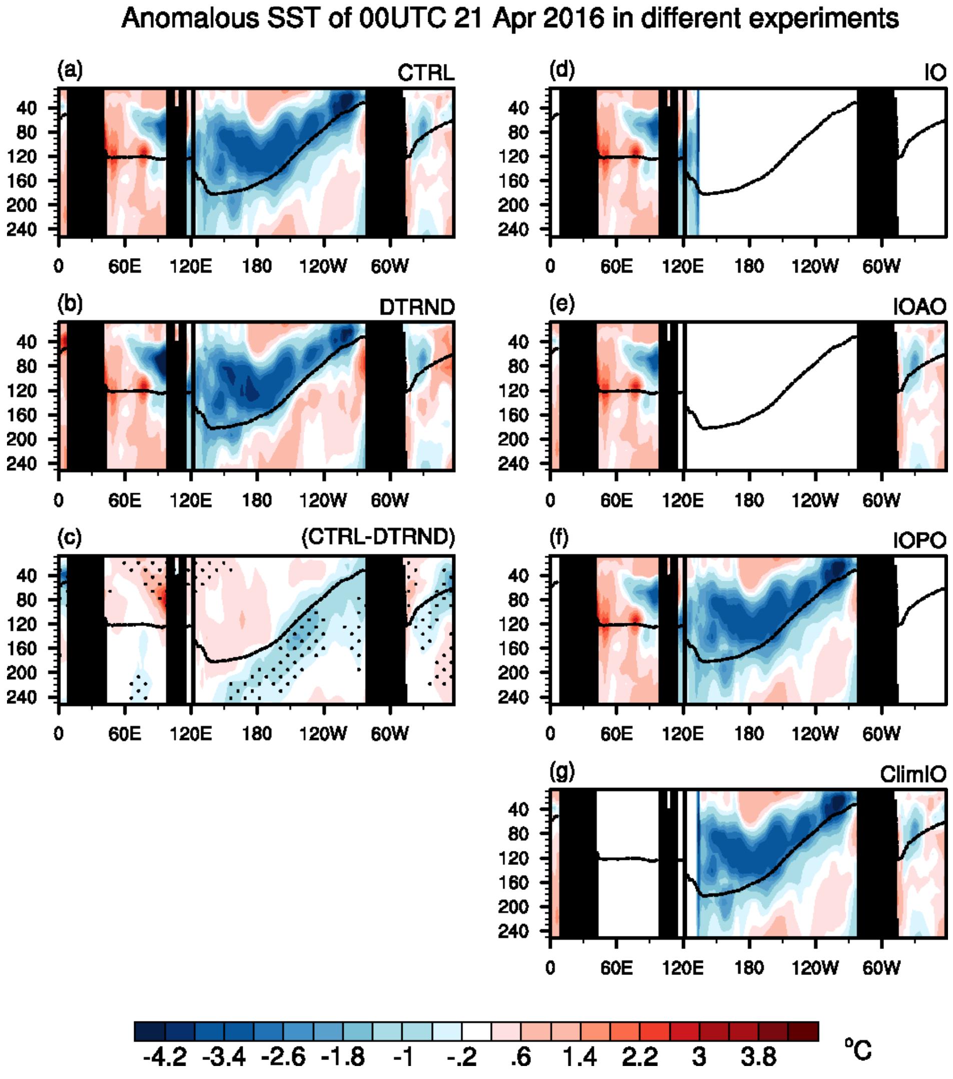

Supplementary Figure S5. Anomaly patterns of PEODAS subsurface temperature on the equator at 00 UTC 21 April 2016, indicating the different initial conditions used for the six forecast experiments. (a)-(g) The same as Figure S4. The black line indicates the climatological depth of 20°C isotherm on forecast day 1 (i.e. 22 April), which was used due to the unavailability of the equivalent data at 00 UTC 21 April 2016. Plots were generated using the NCAR Command Language version 6.3.0 (www.ncl.ucar.edu).

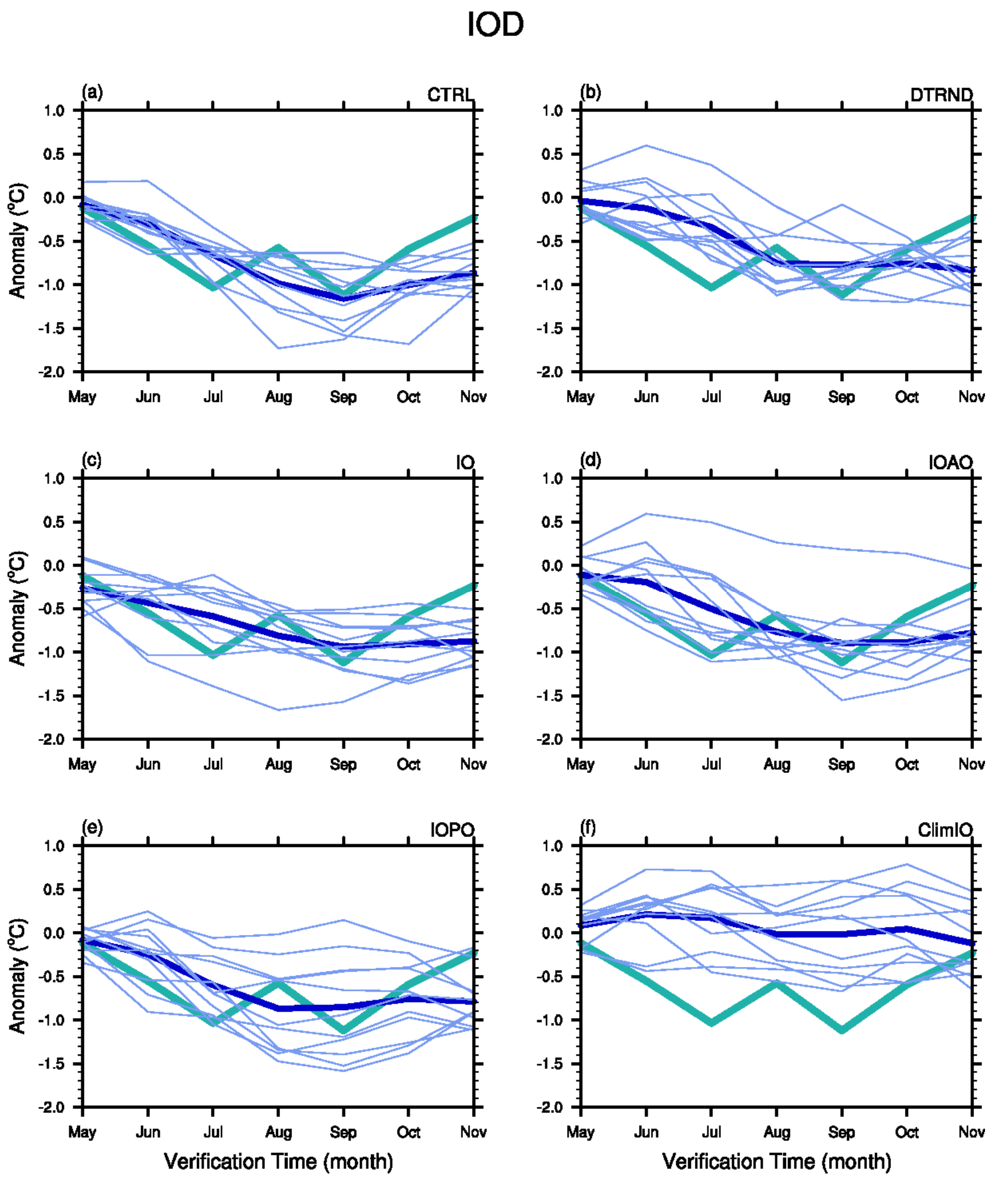

Supplementary Figure S6. Forecast DMI in the six experiments. (a) Forecasts from CTRL, which is the same as Figure 2 (a). (b)-(f) Forecasts from the DTRND, IO, IOAO, IOPO and ClimIO experiments, respectively. The thick blue line and thin blue lines indicate the ensemble mean forecast and the 11 member forecasts of each experiment, respectively. The thick green line denotes the observed DMI index obtained using PEODAS reanalysis. Plots were generated using the NCAR Command Language version 6.3.0 (www.ncl.ucar.edu).

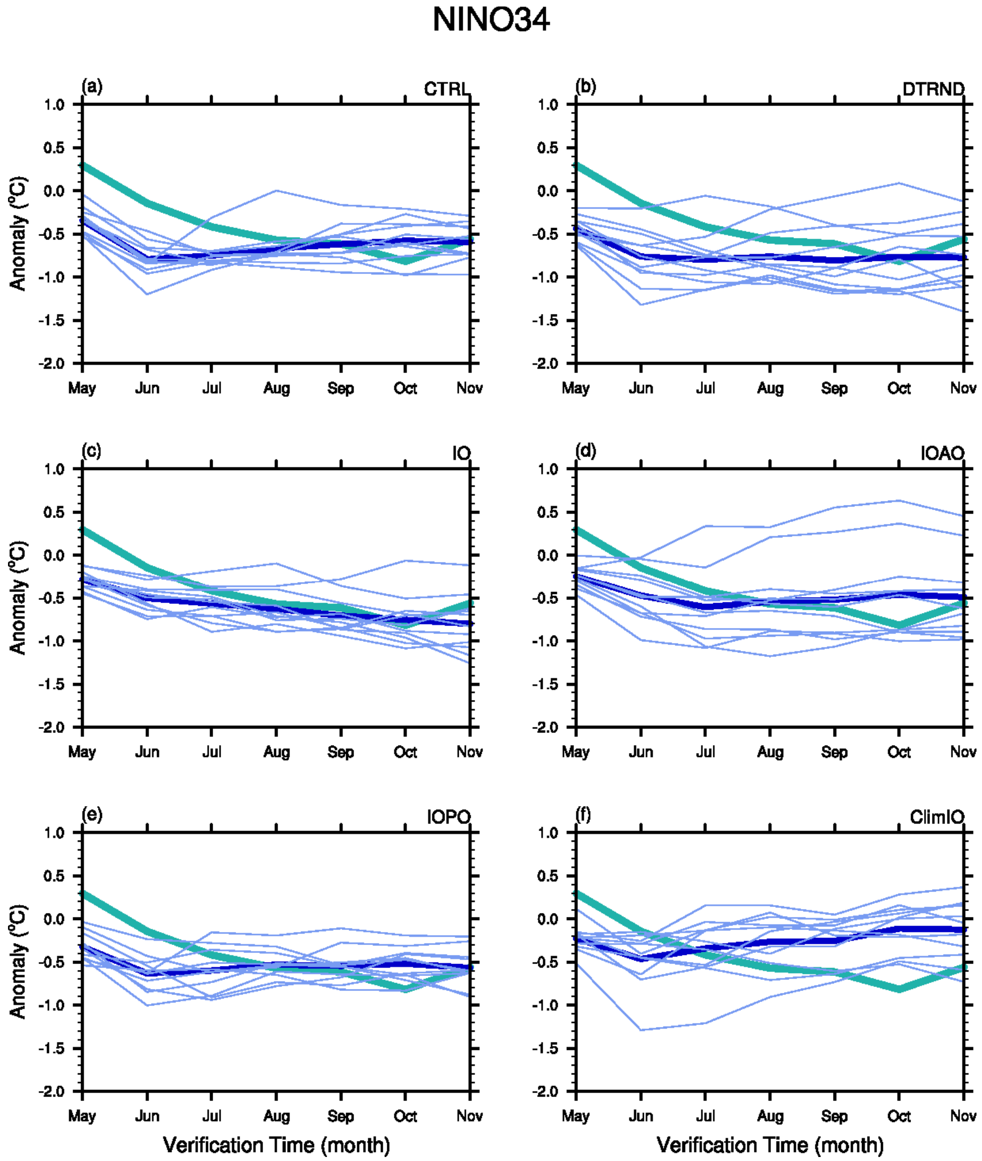

Supplementary Figure S7. Forecast NINO3.4 index in the six experiments. (a) Forecasts from CTRL, which is the same as Figure 2 (c). (b)-(f) Forecasts from the DTRND, IO, IOAO, IOPO and ClimIO experiments, respectively. The thick blue line and thin blue lines indicate the ensemble mean forecast and the 11 member forecasts of each experiment, respectively. The thick green line denotes the observed NINO3.4 index obtained using PEODAS reanalysis. Plots were generated using the NCAR Command Language version 6.3.0 (www.ncl.ucar.edu).

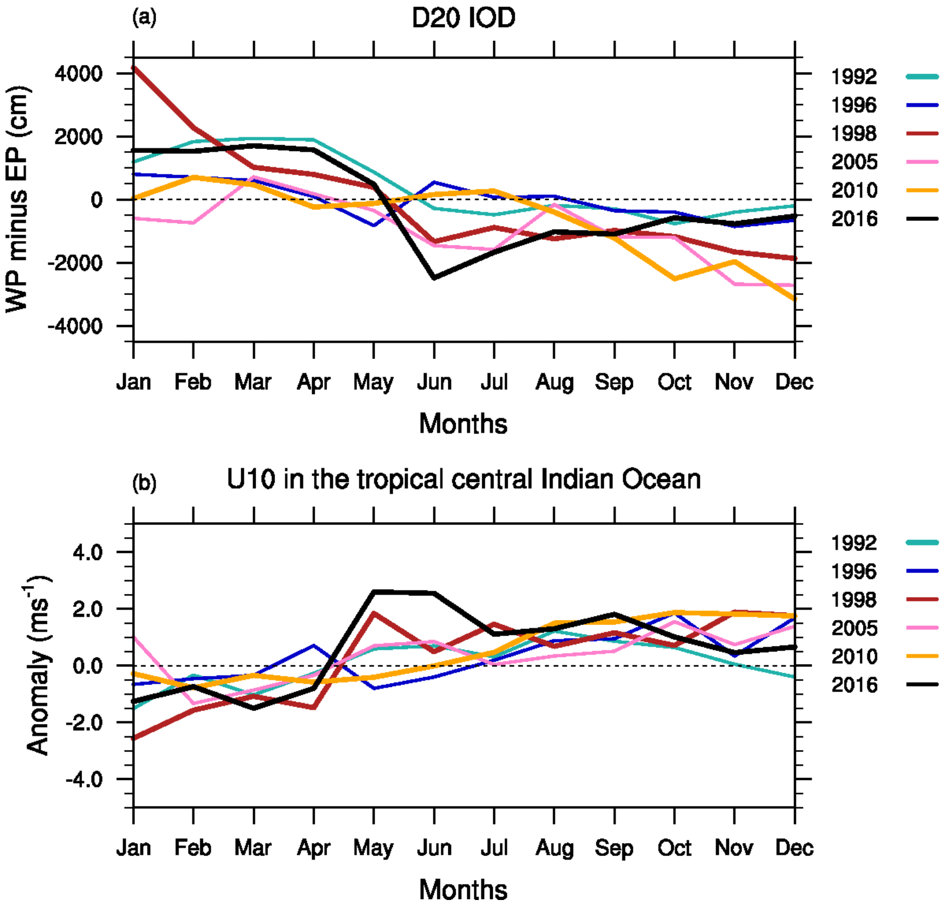

Supplementary Figure S8. Comparison of tropical Indian Ocean subsurface and surface conditions of 2016 to those of the previous five selected years of strong –ve IOD events peaking in June to September. Monthly evolution of de-trended (a) subsurface Indian Ocean Dipole (IOD) and (b) 10 m zonal winds averaged over the tropical central Indian Ocean (5°S-5°N, 50-100°E). Subsurface IOD was computed as the difference of 20°C isotherm depth between the western pole (WP; 10°S-10°N,50-70°E) and the eastern pole (EP; 10°S-0, 90-110°E), following the definition used by Shinoda et al.[6] The positive phase of the subsurface IOD indicates anomalous warming and cooling in the tropical western and eastern Indian Ocean subsurface, respectively, and the negative phase indicates the opposite. Plots were generated using the NCAR Command Language version 6.3.0 (www.ncl.ucar.edu).

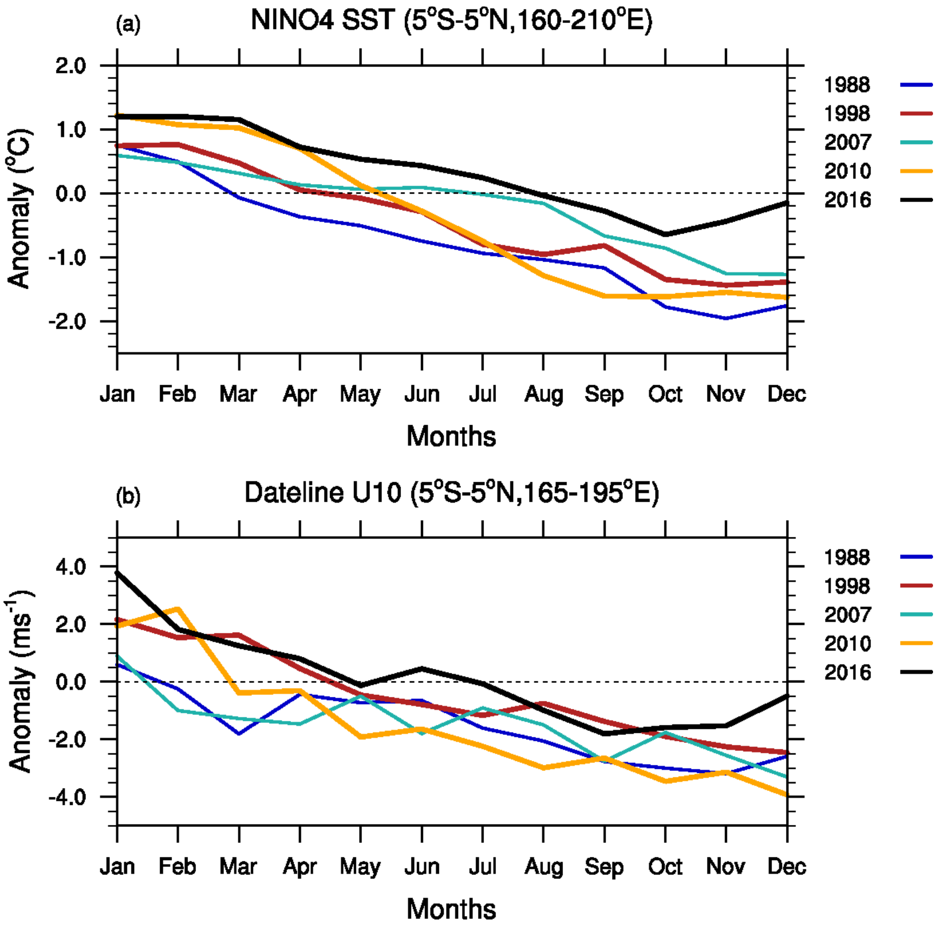

Supplementary Figure S9. Comparison of tropical central Pacific surface conditions of 2016 to those of the previous four selected years of strong La Niña events peaking in November to January. Monthly evolution of (a) NINO4 SST and (b) 10 m zonal wind anomalies averaged over the dateline (5°S-5°N, 165-195°E). Plots were generated using the NCAR Command Language version 6.3.0 (www.ncl.ucar.edu).

Supplementary reference list

1. North, G. R., Bell, T. L., Cahalan, R. F. & Moeng, F. J. Sampling Errors in the Estimation of Empirical Orthogonal Functions. *Mon. Weather Rev.* **110,** 699–706 (1982).

2. Yin, Y., Alves, O. & Oke, P. R. An Ensemble Ocean Data Assimilation System for Seasonal Prediction. *Mon. Weather Rev.* **139,** 786–808 (2011).

3. Rayner, N. A. *et al.* Global analyses of sea surface temperature, sea ice, and night marine air temperature since the late nineteenth century. *J. Geophys. Res.* **108,** 4407 (2003).

4. Reynolds, R. W., Rayner, N. A., Smith, T. M., Stokes, D. C. & Wang, W. An improved in situ and satellite SST analysis for climate. *J. Clim.* **15,** 1609–1625 (2002).

5. Hurrell, J. W., Hack, J. J., Shea, D., Caron, J. M. & Rosinski, J. A new sea surface temperature and sea ice boundary dataset for the community atmosphere model. *J. Clim.* **21,** 5145–5153 (2008).

6. Shinoda, T., Hendon, H. H. & Alexander, M. A. Surface and subsurface dipole variability in the Indian Ocean and its relation with ENSO. *Deep Sea Res. Part I Oceanogr. Res. Pap.* **51,** 619–635 (2004).
